# Therapeutic Potential of *Cajanus cajan* (L.) *Millsp*. Leaf Extract in Modulating Gut Microbiota and Immune Response for the Treatment of Inflammatory Bowel Disease

**DOI:** 10.3390/ph18010067

**Published:** 2025-01-09

**Authors:** Mingzhang Lin, Linghua Piao, Zhendong Zhao, Li Liao, Dayong Wang, Haiwen Zhang, Xiande Liu

**Affiliations:** 1 School of Life and Health Sciences, Hainan Province Key Laboratory of One Health, Collaborative Innovation Center of One Health, Hainan University, No. 58 Renmin Avenue, Haikou 570228, China; 20071000110023@hainanu.edu.cn; 2 Key Laboratory of Tropical Translational Medicine of Ministry of Education & Key Laboratory of Brain Science Research Transformation in Tropical Environment of Hainan Province, School of Basic Medicine and Life Science, Hainan Medical University, Haikou 570228, China; Linghua_piao@hainmc.edu.cn; 3 Analytical & Testing Center, Center for Advanced Studies in Precision Instruments, Hainan University, Haikou 570228, China; zhaozhd898@126.com; 4 School of Tropical Agriculture and Forestry, Hainan University, No. 58 Renmin Avenue, Haikou 570228, China; liaoli@hainanu.edu.cn; 5 School of Pharmaceutical Science, Hainan University, Haikou 570228, China; wangdy@hainanu.edu.cn

**Keywords:** IBD, *Cajanus cajan* leaf extract, inflammatory immune response, gut microbiota

## Abstract

**Background/Objectives:** Inflammatory bowel disease (IBD) is a persistent inflammatory condition affecting the gastrointestinal tract, distinguished by the impairment of the intestinal epithelial barrier, dysregulation of the gut microbiota, and abnormal immune responses. *Cajanus cajan* (L.) *Millsp.*, traditionally used in Chinese herbal medicine for gastrointestinal issues such as bleeding and dysentery, has garnered attention for its potential therapeutic benefits. However, its effects on IBD remain largely unexplored. **Methods:** In this study, the major compounds from *Cajanus cajan* leaf extract (CCLE) were initially characterized by LCMS-IT-TOF. The IBD model was developed in C57BL/6 mice by administering continuous 4% (*w*/*v*) dextran sodium sulfate (DSS) aqueous solution over a period of seven days. The body weight, colon length, disease activity index (DAI), and histopathological examination using hematoxylin and eosin (H&E) staining were performed in the IBD model. The levels of the main inflammatory factors, specifically TNF-α, IL-1β, IL-6, and myeloperoxidase (MPO), were quantified by employing enzyme-linked immunosorbent assay (ELISA) kits. Additionally, the levels of tight junction proteins (ZO-1, Occludin) and oxidative stress enzymes (iNOS, SOD1, CAT) were investigated by qPCR. Subsequently, flow cytometry was employed to analyze the populations of various immune cells within the spleen, thereby assessing the impact of the CCLE on the systemic immune homeostasis of IBD mice. Finally, 16S rDNA sequencing was conducted to examine the composition and relative abundance of gut microbiota across different experimental groups. In addition, molecular docking analysis was performed to assess the interaction between the principal components of CCLE and the aryl hydrocarbon receptor (AHR). **Results:** We identified seven bioactive compounds in CCLE: catechin, cajachalcone, 2-hydroxy-4-methoxy-6-(2-phenylcinyl)-benzoic acid, longistylin A, longistylin C, pinostrobin, amorfrutin A, and cajaninstilbene acid. Our results demonstrated that oral administration of CCLE significantly alleviates gastrointestinal symptoms in DSS-induced IBD mice by modulating the balance of gut-derived pro- and anti-inflammatory cytokines. This modulation is associated with a functional correction in M1/M2 macrophage polarization and the Th17/Treg cell balance in splenic immune cells, as well as shifts in the populations of harmful bacteria (*Erysipelatoclostridium* and *Staphylococcus*) and beneficial bacteria (*Odoribacter*, unidentified *Oscillospiraceae*, *Lachnoclostridium*, and *Oscillibacter*) in the gut. Furthermore, cajaninstilbene acid, longistylin A, and longistylin C were identified as potential AhR agonists. **Conclusions:** The present results suggested that CCLE, comprising stilbenes like cajaninstilbene acid, longistylin A, and longistylin C, protects the epithelial barrier’s structure and function against DSS-induced acute IBD by restoring gut microbiota balance and systemic immune response as AhR agonists. Overall, CCLE represents a promising natural product-based therapeutic strategy for treating IBD by restoring gut microbiota balance and modulating systemic immune responses.

## 1. Introduction

Inflammatory bowel disease (IBD) is a chronic inflammatory disorder characterized by persistent inflammation of the gastrointestinal tract, affecting millions of people worldwide. This disorder is closely linked to disruptions in the intestinal epithelial barrier, imbalances in the gut microbiota, and abnormal immune responses [1,2]. Conventional treatments for IBD, such as corticosteroids, immunomodulators, and antibody agents, are commonly used [3] but often come with significant adverse effects, including diarrhea, hemolytic anemia, osteoporosis, hypoalbuminemia, and granulocytopenia [4].

Given these challenges, there has been growing interest in exploring the therapeutic potential of herbs and natural compounds; notably, Shaoyao [5], taxifolin [6], and ginseng [7] have shown promising effects. Among these, *Cajanus cajan (L.) Millsp*., extensively cultivated in southern China, has a long history in Chinese herbal medicine for treating gastrointestinal ailments [8]. *Cajanus cajan (L.) Millsp*., documented in traditional texts such as “Lu Chuan Ben Cao” and “Quan Zhou Ben Cao” [9], is known for its medicinal properties against ulcers, diarrhea, and dysentery [10,11]. *Cajanus cajan* is rich in flavonoids and chalcones, which have shown potential therapeutic effects on IBD [12,13,14].

Therefore, the aim of this study is to analyze the chemical compounds in *Cajanus cajan* extract and evaluate its effects on DSS-induced IBD in mice.

## 2. Results

### 2.1. The Identification of Bioactive Compounds

**Figure 1:**
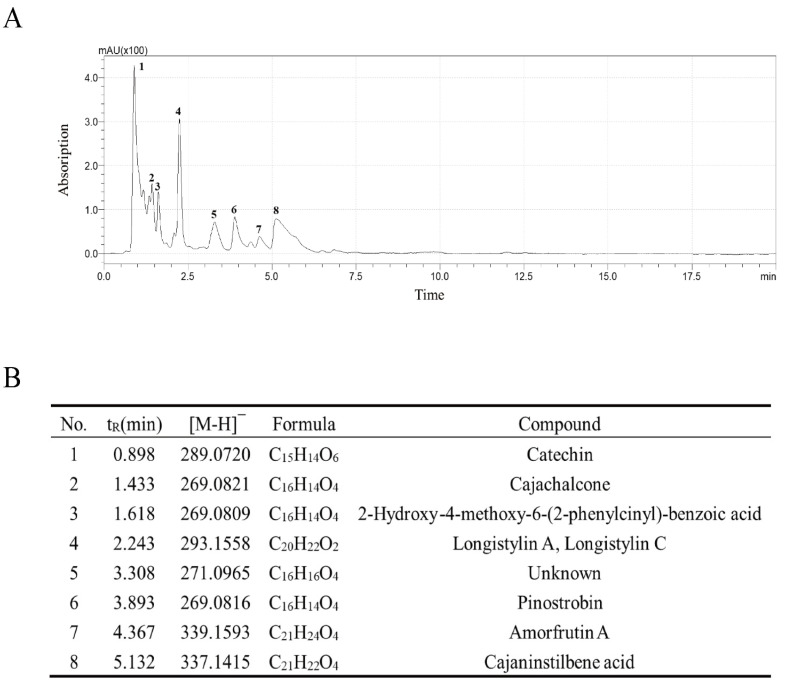
Identification of bioactive compounds in CCLE using LCMS-IT-TOF. (**A**) Eight main peaks separated at a wavelength of 280 nm. (**B**) Seven chemical compounds identified in CCLE.

To evaluate the medicinal potential of CCLE, its bioactive compounds were separated and identified using LCMS-IT-TOF. The analysis revealed the presence of seven chemical compounds: catechin, cajachalcone, 2-hydroxy-4-methoxy-6-(2-phenylcinyl)-benzoic acid, longistylin A, longistylin C, pinostrobin, amorfrutin A, and cajaninstilbene acid (Figure 1A,B).

### 2.2. Therapeutic Efficacy of CCLE Against IBD

**Figure 2:**
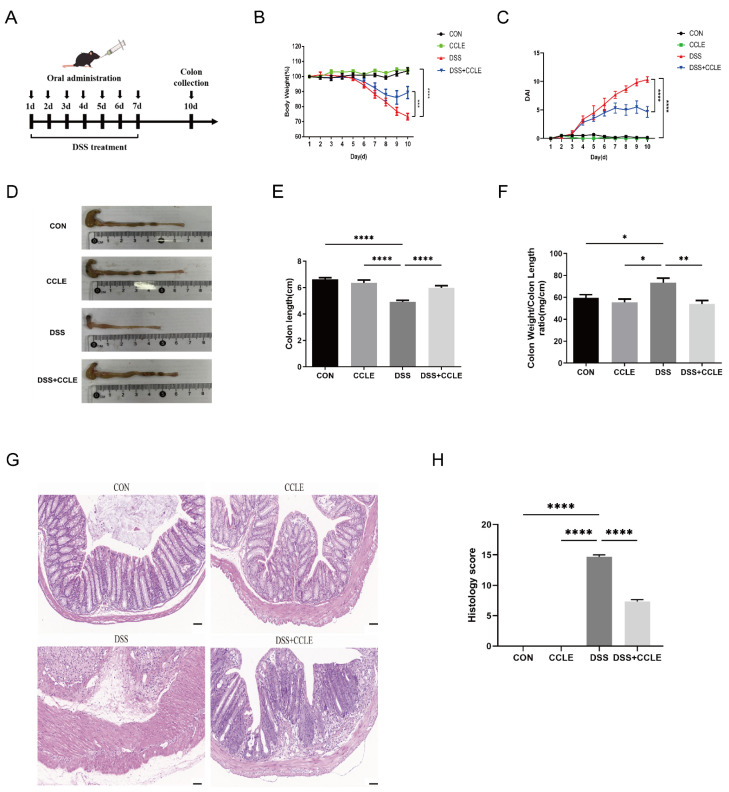
Therapeutic efficacy of CCLE in DSS-induced IBD mice. (**A**) Administration schematic for DSS-induced IBD mice. Quantitative analysis of (**B**) weight changes and (**C**) disease activity index (DAI) in DSS-induced IBD mice. (**D**) Representative images of colons from DSS-induced IBD mice. Quantitative analysis of (**E**) colon length changes and (**F**) colon weight-to-length ratio changes. (**G**) H&E staining. Scale of 50 μm. (**H**) Histological scores of colons (*n* = 3). Data are expressed as mean ± SEM (*n* = 6). * *p* < 0.05, ** *p* < 0.01, *** *p* < 0.001, **** *p* < 0.0001.

To evaluate the therapeutic efficacy of CCLE against IBD, DSS-induced IBD mice were utilized (Figure 2A), displaying symptoms such as weight loss, increased disease activity index (DAI), altered colon weight-to-length ratio, and reduced colon length. The results demonstrated that oral administration of CCLE significantly improved the body weight (*p* < 0.001, Figure 2B), DAI (*p* < 0.0001, Figure 2C), colon length (*p* < 0.0001, Figure 2D,E), and colon weight-to-length ratio (*p* < 0.01, Figure 2F) in DSS-induced IBD mice. Furthermore, histological evaluation of the colon revealed that CCLE administration markedly ameliorated DSS-induced pathological changes, including damaged epithelia, irregular crypt structures, inflammatory cell infiltration, and overall histology score (*p* < 0.0001) (Figure 2G,H). Collectively, these findings suggest that oral administration of CCLE effectively mitigates DSS-induced acute IBD.

### 2.3. Effect of CCLE Administration on Inflammatory Cytokines

**Figure 3:**
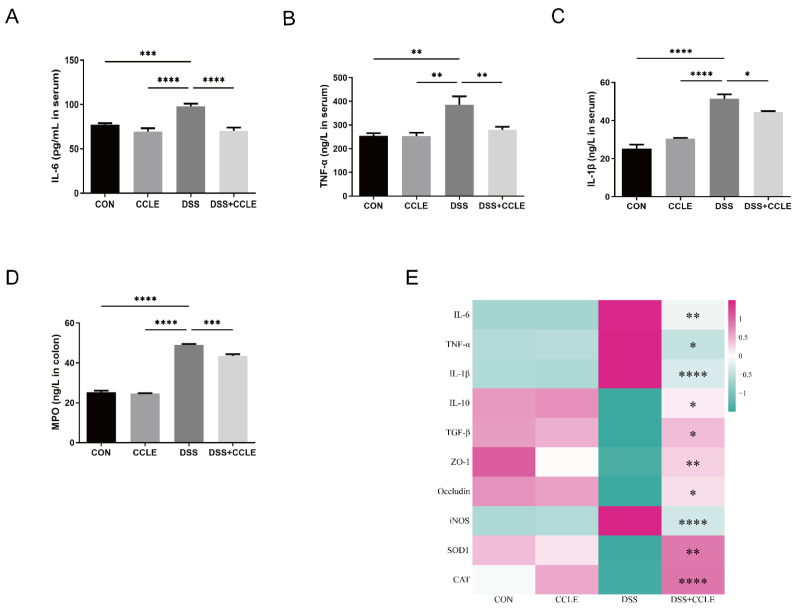
Effect of CCLE on cytokines in DSS-induced IBD mice. Quantitative analysis of (**A**) IL-6, (**B**) TNF-α, and (**C**) IL-1β in serum. (**D**) MPO expression in colon myeloperoxidase (MPO). (**E**) Heatmap of mRNA levels for colon-derived inflammatory cytokines, tight junction proteins, and oxidant enzymes. Data are expressed as mean ± SEM (*n* = 3). * *p* < 0.05, ** *p* < 0.01, *** *p* < 0.001, **** *p* < 0.0001.

To determine whether CCLE mitigates acute IBD through its anti-inflammatory effects, we measured the serum levels of pro-inflammatory cytokines, including IL-6, TNF-α, and IL-1β, as well as colon myeloperoxidase (MPO) using ELISA. The results indicated that CCLE significantly downregulates IL-6 (*p* < 0.0001, Figure 3A), TNF-α (*p* < 0.01, Figure 3B), IL-1β (*p* < 0.05, Figure 3C), and MPO (*p* < 0.001, Figure 3D) in DSS-induced IBD mice. Furthermore, we analyzed pro-inflammatory cytokines (IL-6, TNF-α, IL-1β), anti-inflammatory cytokines (IL-10, TGF-β), tight junction proteins (ZO-1, Occludin), and oxidative stress enzymes (inducible nitric oxide synthase [iNOS], superoxide dismutase [SOD1], catalase [CAT]) in colon tissue using real-time PCR. The findings demonstrated that CCLE downregulates pro-inflammatory cytokines and upregulates anti-inflammatory cytokines (Figure 3E and Figure S1A–F). Additionally, tight junction proteins and antioxidant enzymes, such as SOD1 (*p* < 0.01) and CAT (*p* < 0.0001), were upregulated by CCLE, while the pro-oxidant enzyme iNOS was downregulated (*p* < 0.0001, Figure 3E and Figure S2A–D). Tight junction proteins and oxidative stress enzymes are critical for maintaining epithelial structure and function [15]. Therefore, we conclude that CCLE ameliorates DSS-induced acute epithelial barrier damage by modulating the balance of gut-derived pro- and anti-inflammatory cytokines.

### 2.4. Effect of CCLE Administration on Splenic Immune Response

**Figure 4:**
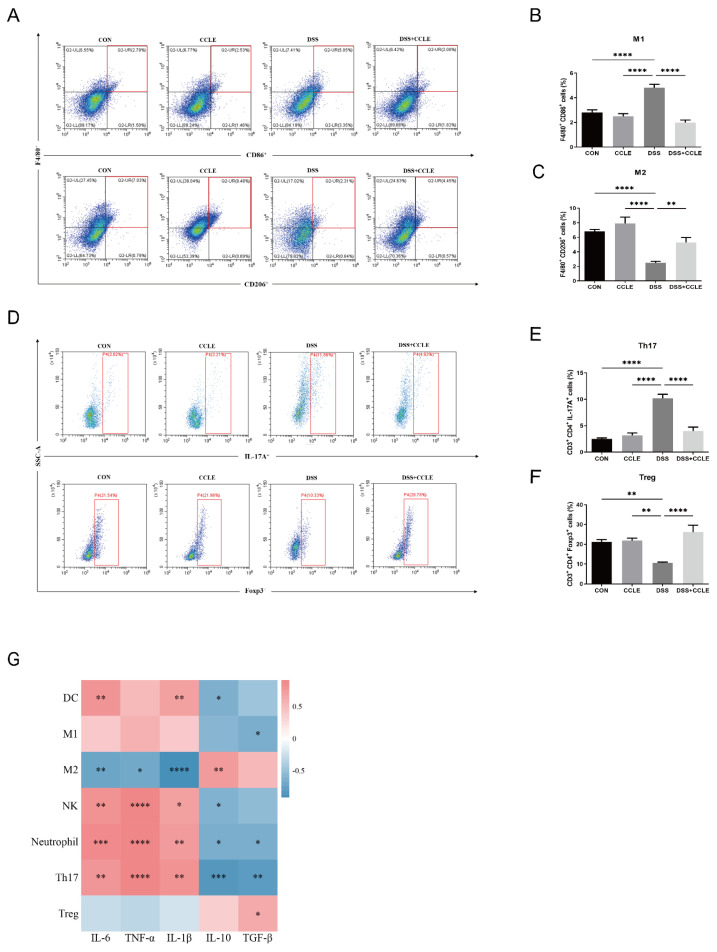
Effect of CCLE on the proportion of splenic immune cells in DSS-induced IBD mice. (**A**) Flow cytometry analysis of M1-like (F4/80+CD86+) and M2-like (F4/80+CD206+) macrophages in splenic immune cells of DSS-induced IBD mice. Quantitative analysis of (**B**) M1-like macrophages (F4/80+CD86+) and (**C**) M2-like macrophages (F4/80+CD206+). (**D**) Flow cytometry analysis of Th17 cells (CD3+CD4+IL-17A+) and Treg cells (CD3+CD4+Foxp3+) in splenic immune cells of DSS-induced IBD mice. Quantitative analysis of (**E**) Th17 cells (CD3+CD4+IL-17A+) and (**F**) Treg cells (CD3+CD4+Foxp3+). (**G**) Spearman’s correlation between colon-derived cytokines and splenic immune cells in DSS-induced IBD mice. Data are expressed as mean ± SEM (*n* = 3). * *p* < 0.05, ** *p* < 0.01, *** *p* < 0.001, **** *p* < 0.0001.

The cross-talk between gut-derived cytokines and splenic immune cells is a critical component of the body’s integrated response to inflammation [16]. To investigate this interaction, we analyzed the proportion of splenic immune cells using flow cytometry. The results demonstrated that CCLE negatively regulates pro-inflammatory immune cells, such as M1-like macrophages (*p* < 0.0001, Figure 4A,B) and Th17 cells (*p* < 0.0001, Figure 4D,E), while positively regulating anti-inflammatory immune cells, such as M2-like macrophages (*p* < 0.01, Figure 4A,C) and Treg cells (*p* < 0.0001, Figure 4D,F) in DSS-induced IBD mice. Additionally, CCLE decreased the levels of other immune cells, including neutrophils (*p* < 0.0001), NK cells (*p* < 0.001), and dendritic cells (DCs, *p* < 0.0001) in splenocytes (Figure S3A–F).

We then conducted pairwise correlation analyses between gut-derived cytokines and the proportion of splenic immune cells. Pro-inflammatory cytokines showed the following correlations: IL-1β positively correlated with DCs (*p* < 0.01), NK cells (*p* < 0.05), neutrophils (*p* < 0.01), and Th17 cells (*p* < 0.01), and negatively correlated with M2-like macrophages (*p* < 0.001); IL-6 positively correlated with DCs (*p* < 0.01), NK cells (*p* < 0.01), neutrophils (*p* < 0.001), and Th17 cells (*p* < 0.01), and negatively correlated with M2-like macrophages (*p* < 0.01); TNF-α positively correlated with NK cells (*p* < 0.001), neutrophils (*p* < 0.001), and Th17 cells (*p* < 0.001), and negatively correlated with M2-like macrophages (*p* < 0.05). Anti-inflammatory cytokines displayed the following correlations: IL-10 negatively correlated with DCs (*p* < 0.05), NK cells (*p* < 0.05), neutrophils (*p* < 0.05), and Th17 cells (*p* < 0.001), and positively correlated with M2-like macrophages (*p* < 0.01); TGF-β negatively correlated with M1-like macrophages (*p* < 0.05), neutrophils (*p* < 0.05), and Th17 cells (*p* < 0.01), and positively correlated with Treg cells (*p* < 0.05). These findings suggest that CCLE modulates the proportion of splenic immune cells by influencing the balance of gut-derived pro- and anti-inflammatory cytokines, thereby attenuating the systemic inflammatory response in DSS-induced IBD mice.

### 2.5. Effect of CCLE Administration on Gut Microbiota

**Figure 5:**
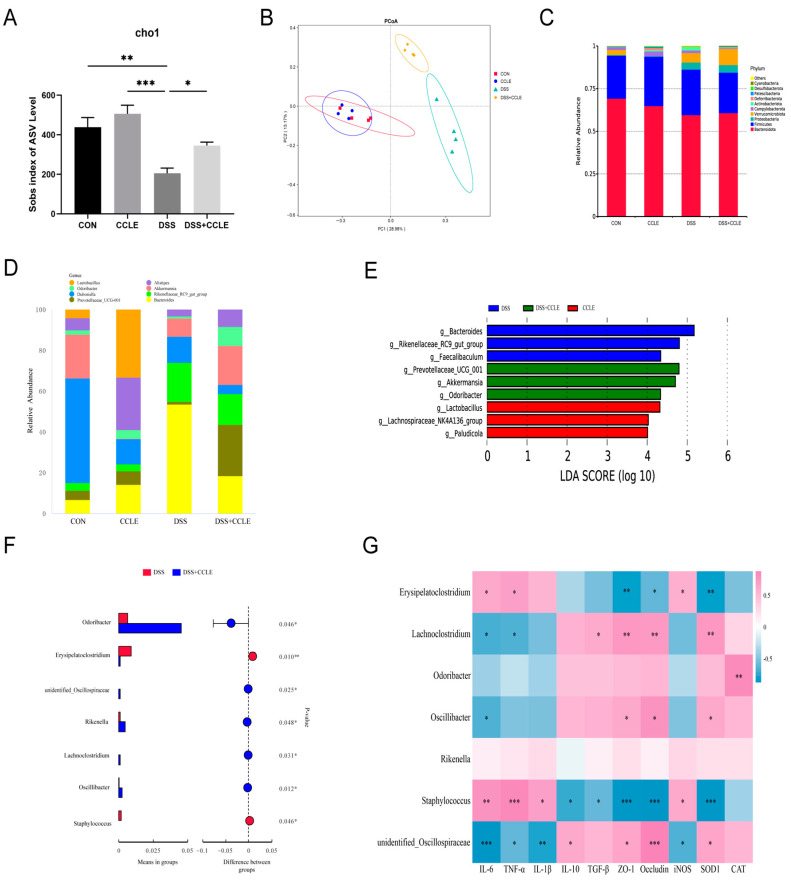
Effect of CCLE on gut microbiota in DSS-induced IBD mice. Quantitative analysis of (**A**) Chao1 index and (**B**) PCoA. Composition of gut microbiota at the (**C**) phylum level and (**D**) genus level. (**E**) LEfSe analysis of gut microbiota. (**F**) Quantitative analysis of microbiota in DSS versus DSS+CCLE groups. (**G**) Spearman’s correlation between gut microbiota and colon-derived cytokines in DSS-induced IBD mice (*n* = 4). * *p* < 0.05, ** *p* < 0.01, *** *p* < 0.001.

Dysbiosis of the gut microbiota can lead to impaired gut barrier structure and function, as certain pathogens and opportunistic bacteria produce toxins and metabolic byproducts. This process promotes the development of gut inflammation in inflammatory bowel disease (IBD) [17]. To investigate this, we analyzed the gut microbiota using 16S rRNA sequencing of fecal samples from DSS-induced IBD mice. The Sobs index at the ASV level revealed that CCLE significantly mitigates the DSS-induced decline in microbial diversity and richness (Figure 5A). PCoA analysis showed significant differences in gut microbial community composition between the DSS and DSS+CCLE groups (Figure 5B). At the phylum level, the dominant families were *Bacteroidetes*, *Firmicutes*, and *Proteobacteria* (Figure 5C), while at the genus level, *Bacteroidetes*, *Prevotellaceae_UCG-001*, and *Rikenellaceae_RC9_gut_group* were predominant (Figure 5D).

LEfSe (Linear Discriminant Analysis Effect Size) analysis indicated that *Bacteroides*, *Rikenellaceae_RC9_gut_group*, and *Faecalibaculum* were dominant in the DSS group; *Prevotellaceae_UCG-001*, *Akkermansia*, and *Odoribacter* were dominant in the DSS+CCLE group; and *Lactobacillus*, *Lachnospiraceae_NK4A136_group*, and *Paludicola* were dominant in the CCLE group (Figure 5E). Additionally, CCLE significantly reduced the abundance of *Odoribacter* (*p* = 0.046), *unidentified_Oscillospiraceae* (*p* = 0.025), *Rikenella* (*p* = 0.048), *Lachnoclostridium* (*p* = 0.031), and *Oscillibacter* (*p* = 0.012) in DSS-induced IBD mice, while increasing the abundance of *Erysipelatoclostridium* (*p* = 0.010) and *Staphylococcus* (*p* = 0.046, Figure 5F).

Spearman’s correlation analysis between gut-derived cytokines, tight junction proteins, oxidant enzymes, and gut microbiota revealed significant associations: *Erysipelatoclostridium* was positively correlated with IL-6 (*p* < 0.05), TNF-α (*p* < 0.05), and iNOS (*p* < 0.05), but negatively correlated with Occludin (*p* < 0.05), ZO-1 (*p* < 0.01), and SOD1 (*p* < 0.01); *Lachnoclostridium* was positively correlated with TGF-β (*p* < 0.05), ZO-1 (*p* < 0.01), Occludin (*p* < 0.01), and SOD1 (*p* < 0.01), but negatively correlated with IL-6 (*p* < 0.01) and TNF-α (*p* < 0.01); *Odoribacter* was positively correlated with CAT (*p* < 0.01); *Oscillibacter* was positively correlated with Occludin (*p* < 0.05), ZO-1 (*p* < 0.05), and SOD1 (*p* < 0.05), but negatively correlated with IL-6 (*p* < 0.05); *Staphylococcus* was positively correlated with IL-6 (*p* < 0.01), TNF-α (*p* < 0.001), IL-1β (*p* < 0.05), and iNOS (*p* < 0.05), but negatively correlated with IL-10 (*p* < 0.05), TGF-β (*p* < 0.05), ZO-1 (*p* < 0.001), Occludin (*p* < 0.001), and SOD1 (*p* < 0.001, Figure 5G); *unidentified_Oscillospiraceae* was negatively correlated with IL-6 (*p* < 0.001), IL-1β (*p* < 0.05), TNF-α (*p* < 0.001), and iNOS (*p* < 0.05), but positively correlated with IL-10 (*p* < 0.05), ZO-1 (*p* < 0.05), Occludin (*p* < 0.001), and SOD1 (*p* < 0.05). These results suggest that CCLE modulates gut-derived inflammatory cytokines by balancing beneficial and harmful bacteria in the gut microbiota, potentially counteracting DSS-induced acute IBD.

### 2.6. CCLE Administration Activates AHR-Nrf2-NQO1 Axis

**Figure 6:**
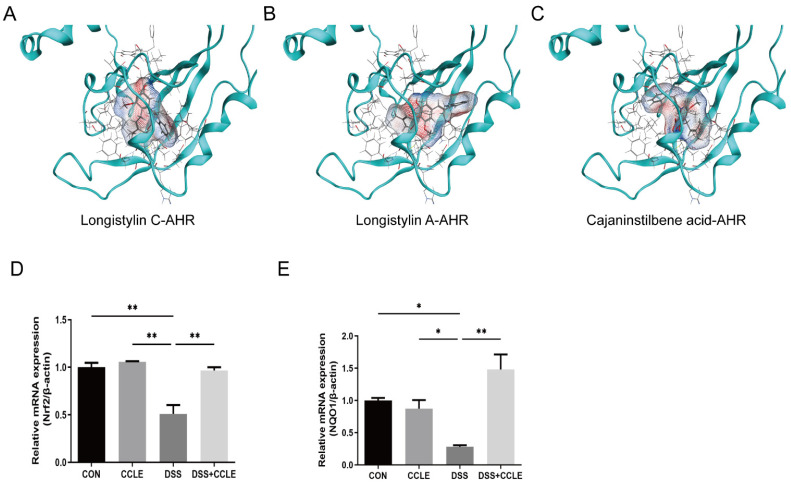
Chemical structures of certain compounds in CCLE and their interaction with AHR protein structure. Molecular docking simulation images for (**A**) longistylin C and AHR, (**B**) longistylin A and AHR, and (**C**) cajaninstilbene acid and AHR. Quantitative analysis of mRNA levels of (**D**) Nrf2 and (**E**) NQO1 in colon tissues. Data are expressed as mean ± SEM (*n* = 3). * *p* < 0.05, ** *p* < 0.01.

The aryl hydrocarbon receptor (AHR) is pivotal in the pathogenesis and treatment of inflammatory bowel disease (IBD) due to its diverse immunomodulatory effects, which include immune regulation, modulation of the gut microbiota, anti-inflammatory actions, and tissue repair [18]. To determine whether specific compounds in *Cajanus cajan* leaf extract (CCLE) function as AHR agonists in IBD, we conducted molecular docking studies using the three-dimensional (3D) crystal structure of AHR and seven chemical structures identified in CCLE by LCMS-IT-TOF. Our findings revealed that longistylin C (−6.6343 kcal/mol), longistylin A (−6.0048 kcal/mol), and cajaninstilbene acid (−5.7876 kcal/mol) effectively bind to the AHR protein, as indicated by their respective binding energies (Figure 6A–C). Following this, we examined the downstream genes of AHR, specifically Nrf2 and NQO1, using real-time PCR. The results showed that CCLE upregulates the mRNA levels of Nrf2 and NQO1 (Figure 6D,E). These findings suggest that longistylin C, longistylin A, and cajaninstilbene acid from CCLE are potent AHR agonists. Their activity is associated with enhanced immune response, modulation of the gut microbiota, anti-inflammatory effects, and tissue repair in DSS-induced IBD mice.

## 3. Discussion

In this study, we identified seven bioactive compounds in CCLE, including catechin, cajachalcone, 2-hydroxy-4-methoxy-6-(2-phenylcinyl)-benzoic acid, longistylin A, longistylin C, pinostrobin, amorfrutin A, and cajaninstilbene acid. Specifically, cajachalcone and pinostrobin [19], as flavonoids, and longistylin A [20], longistylin C, and cajaninstilbene acid [21], as stilbenes, exhibit notable anti-inflammatory and antioxidant properties. We hypothesize that these compounds contribute significantly to the therapeutic potential of CCLE against gastrointestinal disorders.

To test this hypothesis, we first demonstrated that oral administration of CCLE exerts a significant protective effect on gastrointestinal symptoms in DSS-induced IBD mice by modulating the balance of anti- and pro-inflammatory cytokines derived from the colon. This finding aligns with previous studies indicating that flavonoids and stilbenes extracted from Cajanus cajan leaves possess significant anti-inflammatory activities [22,23].

Inflammatory cytokines produced in the gut can enter the bloodstream, reaching the spleen and influencing the function of splenic immune cells, thereby amplifying the inflammatory response [24]. We demonstrated that CCLE administration regulates the balance between M1-like and M2-like macrophages and between Th17 and Treg cells in the spleens of DSS-induced IBD mice. Additionally, it decreases the proportions of neutrophils, NK cells, and dendritic cells. Pairwise correlation analysis further revealed functional relationships, with M2-like macrophages and Treg cells correlating with anti-inflammatory cytokines (e.g., TGF-β) and M1-like macrophages and Th17 cells correlating with pro-inflammatory cytokines (e.g., IL-6, TNF-α, IL-1β). Modulating macrophage polarization from the M1 to M2 phenotype and shifting towards Treg cell dominance, or reducing Th17 cells, are potential therapeutic strategies for IBD [25].

Moreover, we demonstrated that CCLE administration significantly decreases the abundance of harmful bacteria such as *Erysipelatoclostridium* and *Staphylococcus*, while increasing beneficial bacteria such as *Odoribacter*, *unidentified Oscillospiraceae*, *Rikenella*, *Lachnoclostridium*, and *Oscillibacter* in DSS-induced IBD mice. These results are consistent with studies showing that flavonoids and stilbenes modulate the gut microbiota by promoting beneficial bacteria and inhibiting harmful bacteria [17]. Pairwise correlation analysis indicated that harmful bacteria positively correlate with pro-inflammatory cytokines and pro-oxidant enzymes, while negatively correlating with anti-inflammatory cytokines, tight junction proteins, and antioxidant enzymes. Conversely, beneficial bacteria negatively correlate with pro-inflammatory cytokines and positively with anti-inflammatory cytokines and tight junction proteins. Prior studies have shown that beneficial bacteria such as *Odoribacter* [26], *Lachnoclostridium*, and *Oscillibacter* produce short-chain fatty acids, which are crucial for maintaining the structure and function of the epithelial barrier by regulating tight junction proteins and anti-inflammatory cytokines in the gut [27,28]; harmful bacteria such as *Erysipelatoclostridium* [29,30] and *Staphylococcus* [31] are elevated in the feces of patients with IBD.

Further, our study demonstrated that stilbenes such as cajaninstilbene acid, longistylin A, and longistylin C were identified as potential AhR agonists. This observation is supported by previous studies suggesting that activation of AhR can attenuate IBD symptoms by restoring gut microbiota balance and systemic immune response [32]. Resveratrol, a stilbene, shares a similar chemical structure with longistylin A, longistylin C, and cajaninstilbene acid. Previous studies have reported that double-blind randomized clinical trials demonstrated resveratrol supplementation (500 mg/day) improves clinical colitis activity and quality of life in patients with mild-to-moderate ulcerative colitis [33,34]. Additionally, resveratrol has been reported to regulate the Treg/Th17 balance, downregulate pro-inflammatory cytokines, and upregulate anti-inflammatory cytokines in DSS-induced IBD mice [35]. Another study also reported that resveratrol alleviates DSS-induced IBD in mice by regulating the intestinal microbiota–macrophage axis [36]. These observations are very similar to the effects of *Cajanus cajan* extract on DSS-induced IBD mice. A limitation of this study is the lack of evaluation of the effects of individual compounds, such as longistylin A, longistylin C, or cajaninstilbene acid, on IBD in mice. Therefore, our future research will focus on determining the effects of these individual compounds on IBD in mice and developing a precise extraction method for *Cajanus cajan* or synthesizing these compounds.

In summary, this study demonstrated that oral CCLE inhibited DSS-induced IBD symptoms and colonic damage, reduced intestinal inflammation, restored intestinal barrier integrity, and maintained immune homeostasis. Meanwhile, CCLE regulated the structure of the gut microbiota and reduced the relative abundance of potentially harmful bacteria (*Erysipelatoclostridium* and *Staphylococcus*) and enriched the relative abundance of potentially beneficial bacteria (*Odoribacter*, unidentified *Oscillospiraceae*, *Lachnoclostridium*, and *Oscillibacter*). Furthermore, cajaninstilbene acid, longistylin A, and longistylin C were identified as potential AhR agonists. These results suggest that CCLE may be used as a dietary component in the prevention and treatment of colitis.

## 4. Materials and Methods

### 4.1. Preparation of Plant Extracts

The leaves of *Cajanus cajan* were gathered from Hainan University (Haikou, China) with no additional permissions required for sample collection. Dried leaves were ground and sieved through an 80-mesh sieve, followed by extraction with 80% ethanol using ultrasound-assisted techniques at 50 °C for 30 min. Subsequently, the solvent was eliminated via rotary evaporation, yielding a dry extract. This extract was dissolved in ethyl acetate and subjected to further solvent removal through rotary evaporation followed by freeze-drying. The resulting product, designated *Cajanus cajan* leaf extract (CCLE), was obtained.

### 4.2. Identification Compounds in CCLE by LCMS-IT-TOF

CCLE was dissolved in methanol and filtered through a 0.22 μm PTFE microporous filter membrane to obtain the sample solution. Liquid chromatography coupled with mass spectrometry (LCMS-IT-TOF, Shimadzu, Kyoto, Japan) analysis was conducted under the following conditions: LC-30AD liquid chromatograph equipped with a PDA diode array detector, an ODS-C18 column (250 mm × 4.6 mm, 5 μm), and a mobile phase consisting of aqueous 0.1% formic acid (A) and acetonitrile (B). The gradient elution program was as follows: 0–10 min, 80% A; 10–15 min, 80–70% A; 15–16 min, 70–30% A; 16–19 min, 30% A; 19–20 min, 30% A; 20–20.1 min, 80% A. Mass spectrometry operated under positive and negative ionization modes, with electrospray ionization (ESI) source voltages set at 3.5 kV and −3.5 kV, respectively. The nebulizing gas was nitrogen at a flow rate of 1.5 L/min, and argon was used as the collision gas. The heat block and CDL temperatures were maintained at 200 °C. The primary and secondary mass spectrometry (MS) scanning ranged from 100 to 400 *m*/*z*, with an ion accumulation time of 15 ms for MS/MS. Collision-induced dissociation (CID) collision energy was set to 50%, with a collision gas energy also set to 50%.

### 4.3. Induction of IBD Mice Model and Drug Administration

Male C57BL/6 mice (5 weeks old, 20 ± 2 g) were obtained from SiPeiFu (Beijing) Biotechnology Co.(Beijing, China). They were kept with a 12 h light/dark cycle at about 25 °C with free access to pathogen-free food and tap water. All animal procedures were approved by the Animal Ethics Committee of Hainan University. Mice were randomly assigned to four groups with six in each group. The experimental design was developed based on previous studies, incorporating several modifications [37]. As shown in Figure 2A, 4% DSS (MP Biomedical, Santa Ana, CA, USA) solution was administrated to induce experimental IBD through drinking freely for 7 days and followed by sterile distilled water alone for another 3 days. The experimental groups were as follows: (1) CON group: distilled water for 10 days, daily oral administration of PBS for 7 days; (2) CCLE group: distilled water for 10 days, daily oral administration of CCLE (100 mg/kg) for 7 days; (3) DSS group: 4% DSS for 7 days, daily oral administration of PBS for 7 days; (4) DSS+CCLE group: 4% DSS for 7 days, daily oral administration of CCLE (100 mg/kg) for 7 days. Body weights, stool consistency, and rectal bleeding were recorded once daily. Mice were euthanized at day 10 and the colon was excised and washed with PBS solution. The excised colon length was measured and photographed and then stored at −80 °C for further analysis.

### 4.4. Disease Activity Index (DAI)

The disease activity index (DAI) was calculated based on measurements of body weight loss, stool consistency, and rectal bleeding, as previously described [38], scored as follows: body weight loss (0 points = none; 1 point = 1–5% loss; 2 points = 5–10% loss; 3 points = 10–20% loss; 4 points = over 20% loss); stool consistency (0 points = normal; 1 point = soft stools; 2 points = loose stools; 3 points = watery diarrhea); rectal bleeding (0 points = no bleeding; 1 point = slight bleeding; 2 points = bleeding; 3 points = gross bleeding; 4 points = rectal bleeding). The sum of these scores constituted the DAI.

### 4.5. Histological Analysis (H&E Staining)

Colon tissue samples from the mouse IBD model were preserved in a 4% paraformaldehyde solution, subsequently embedded in paraffin, sectioned, and stained utilizing H&E staining kit (Servicebio, Wuhan, China) according to standard protocols, and observed under a light microscope (Nikon Eclipse E100; Nikon, Tokyo, Japan). Histopathological changes were recorded using an imaging system (Nikon DS-U3; Nikon, Japan) and quantitatively analyzed based on previous studies [37]. Briefly, the total score was calculated from the inflammation severity score (0–4), the lesion depth score (0–4), the crypt damage score (0–4), and the lesion range score (0–4).

### 4.6. Enzyme-Linked Immunosorbent Assay (ELISA) Analysis

Colon and serum samples were collected from mouse IBD models to measure the protein levels of MPO (XY9M0010), IL-6 (XY9M0121), IL-1β (XY9M0109), and TNF-α (XY9M0183). ELISA kits were purchased from X-Y Biotechnology (Shanghai, China) and used according to the manufacturer’s instructions. The OD values at 450 nm were measured by Multiskan™ FC Microplate Photometer (Thermo Scientific, Waltham, MA, USA).

### 4.7. Quantitative PCR Analysis

Total RNA from the colon was isolated using an RNA extraction kit (Servicebio, China). cDNA synthesis was performed with the SweScript All-in-One RT kit (Servicebio, China). Quantitative PCR (qPCR) was conducted using the LightCycler 96 PCR System with the SYBR Green Premix Pro Taq HS qPCR kit (Accurate Biology, Changsha, China), following the manufacturer’s protocol. The primers used in this study are listed in Table S1. Gene expression was normalized to β-actin and calculated using the 2^−ΔΔCt^ method.

### 4.8. Flow Cytometry Analysis

Splenocytes isolated from IBD mice were incubated with fluorochrome-labeled antibodies (FITC Anti-Mouse Ly-6G (E-AB-F1108C), APC Anti-Mouse CD86 (E-AB-F0994E), APC Anti-Mouse CD206 (E-AB-F1135E), FITC Anti-Mouse CD80 (E-AB-F0992C), FITC Anti-Mouse F4/80 (E-AB-F0995C), PerCP/Cyanine5.5 Anti-Mouse CD11c (E-AB-F0991J), FITC Anti-Mouse CD161/NK1.1 (E-AB-F0987C), APC Anti-Mouse CD3 (E-AB-F1013E), PE Anti-Mouse CD4 (E-AB-F1353D), FITC Anti-Mouse IL-17A (E-AB-F1272C), and FITC Anti-Mouse Foxp3 (E-AB-F1238C)). All antibodies were purchased from Elabscience (Wuhan, China) and used at a 1:20 dilution. The samples were analyzed using a CytoFLEX Flow Cytometer (Beckman, Brea, CA, USA) and FlowJo software (v10.6.2, TreeStar, Inc., Ashland, OR, USA). At least 10,000 events were analyzed per sample.

### 4.9. Gut Microbiota Analysis

Fecal samples from IBD mice were harvested, frozen in liquid nitrogen, and sent to Novogene Bioinformatics Co., Ltd. (Beijing, China) for microbiota analysis using a 16S sequencing assay. Microbiome DNA was isolated from the fecal samples of mice utilizing the QIAamp^®^ Fast DNA Stool Mini Kit (Qiagen, Dusseldorf, Germany), and 16S rRNA libraries were constructed with QuantiT™ dsDNA HS reagent on an Illumina HiSeq 2500 instrument (Illumina, San Diego, CA, USA). The sequencing data for the 16S rRNA microbiome were analyzed using the NovoMagic platform (https://magic.novogene.com).

### 4.10. Molecular Docking

Molecular docking studies were conducted using the three-dimensional (3D) crystal structure of AHR and chemical structures of selected compounds. The 3D crystal structure of AHR (PDB ID: 5V0L) and the chemical structures of longistylin C (Compound CID: 6446720), longistylin A (Compound CID: 131752397), and cajaninstilbene acid (Compound CID: 9819225) were obtained from the Protein Data Bank and PubChem, respectively. The lowest potential energy conformation was determined by protonating the 3D model in different states of the terminal amide, hydroxyl, thiol, histidine, and titratable groups using MOE software (Molecular Operating Environment, v2019.0102; Chemical Computing Group, Inc., Montreal, QC, Canada).

### 4.11. Statistical Analyses

Statistical analyses were conducted using GraphPad Prism version 9.0. The data were expressed as the mean ± standard error after at least 3 technical replicates. *T*-test was used to compare the mean differences between two groups; one-way ANOVA followed by Dunnett’s multiple comparison test were used to assess the statistical significance of differences between experimental groups; significance levels were indicated as follows: * *p* < 0.05, ** *p* < 0.01, *** *p* < 0.001, **** *p* < 0.0001.

## 5. Conclusions

Our study illustrates that CCLE, comprising stilbenes like cajaninstilbene acid, longistylin A, and longistylin C, protects the epithelial barrier’s structure and function against DSS-induced acute IBD by restoring gut microbiota balance and systemic immune response as AhR agonists. Consequently, CCLE represents a promising natural product-based therapeutic strategy for treating IBD.

## Data Availability

The data presented in this study are available on request from the corresponding author.

## Supplementary Materials

The following supporting information can be downloaded at: https://www.mdpi.com/article/10.3390/ph18010067/s1, Figure S1: Effect of CCLE on cytokine. Figure S2: Effect of CCLE on Tight Junction Proteins and Oxidant Enzymes. Figure S3: Effect of CCLE on splenic immune cells of DSS-induced IBD mice. Table S1: Primers for qRT-PCR.

## Abbreviations

AHR, aryl hydrocarbon receptor; ASV, Amplicon Sequence Variant; CAT, catalase; CCLE, *Cajanus cajan* leaf extract; DAI, disease activity index; DC, dendritic cells; DSS, dextran sodium sulfate; ELISA, enzymelinked immunosorbent assay; H&E, hematoxylin and eosin; IBD, inflammatory bowel disease; IL-10, Interleukin 10; IL-1β, interlenkin 1β; IL-6, interleukin 6; LEfSe, Linear Discriminant Analysis Effect Size; M1, type-1 macrophage; M2, type-II macrophage; MPO, myeloperoxidase; NK, natural killer cell; NQO1, NAD(P)H:quinone oxidoreductase 1; Nrf2, nuclear factor erythroid 2-related factor 2; PCoA, Principal Coordinate Analysis; qPCR, quantitative PCR; SOD1, superoxide dismutase 1; TGF-β, transforming growth factor-β; Th17, T helper cell 17; TNF-α, tumor necrosis factor α; Treg, regulatory T cells; ZO-1, zonula occludens-1.
