# Supplementary material for: Therapeutic Potential of Cajanus cajan (L.) Millsp. Leaf Extract in Modulating Gut Microbiota and Immune Response for the Treatment of Inflammatory Bowel Disease
Source: Pharmaceuticals (Basel). 2025 Jan 9;18(1):67. doi: 10.3390/ph18010067 (PMC11769518; doi:10.3390/ph18010067)
Supplement: Supplementary file 1 [file pharmaceuticals-18-00067-s001.zip › pharmaceuticals-3390828-supplementary.pdf]

This file includes:

I. Supplemental Figures

Supplemental Figure S1 to S3

II. Supplemental Tables

Supplemental Table S1

I. Supplemental Figures

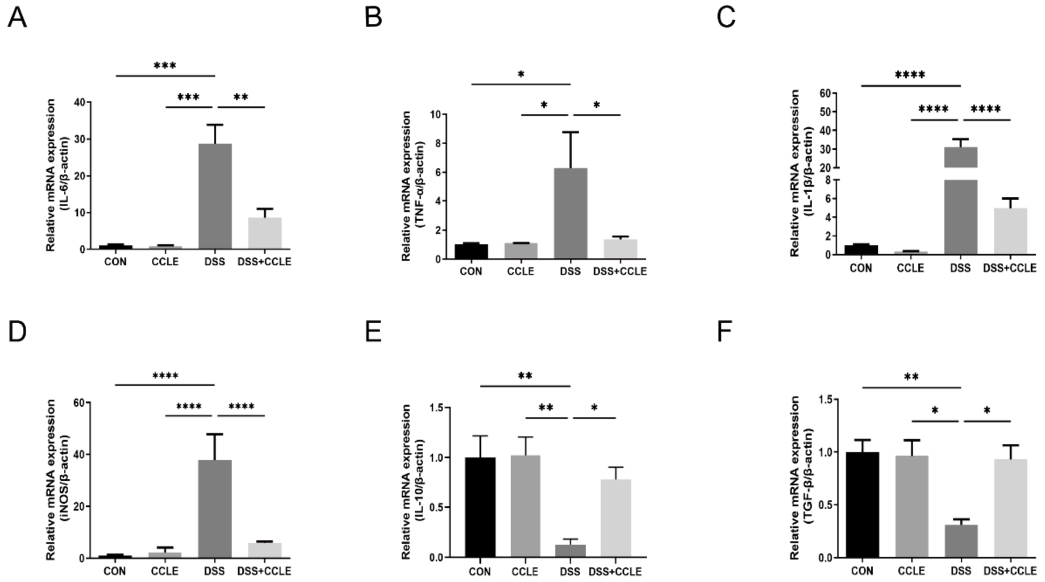

Supplemental Figure S1. Effect of CCLE on cytokine. Quantitative analysis of mRNA levels of (**A**) IL-6, (**B**) TNF-α, (**C**) IL-1β, (**D**) iNOS, (**E**) IL-10, and (**F**) TGF-β in colon tissues. Data are expressed as mean ± SEM ($n = 3$). *$p < 0.05$, **$p < 0.01$, ***$p < 0.001$, ****$p < 0.0001$.

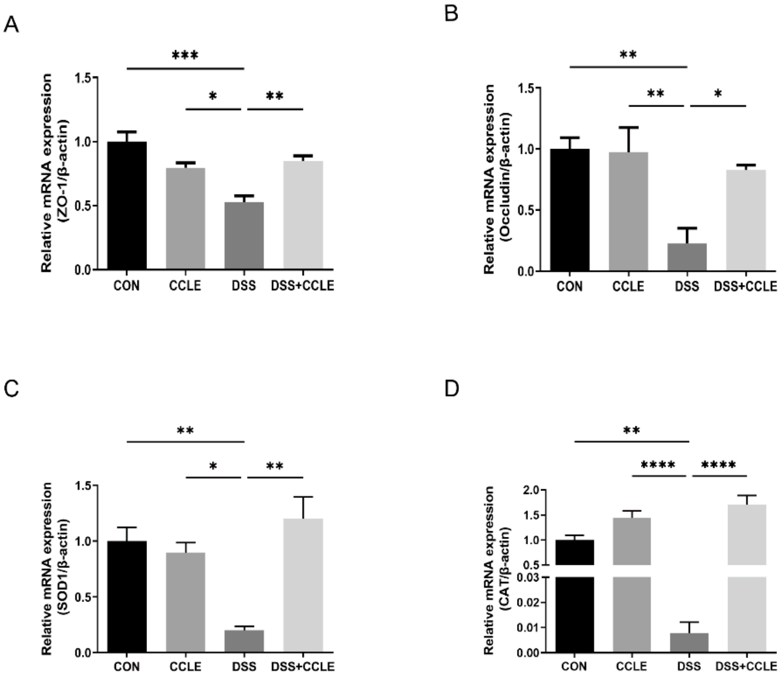

Supplemental Figure S2. Effect of CCLE on Tight Junction Proteins and Oxidant Enzymes. Quantitative analysis of mRNA levels of (**A**) ZO-1, (**B**) Occludin, (**C**) SOD1, and (**D**) CAT in colon tissues. Data are expressed as mean ± SEM (*n* = 3). *$p < 0.05$, **$p < 0.01$, ***$p < 0.001$, ****$p < 0.0001$.

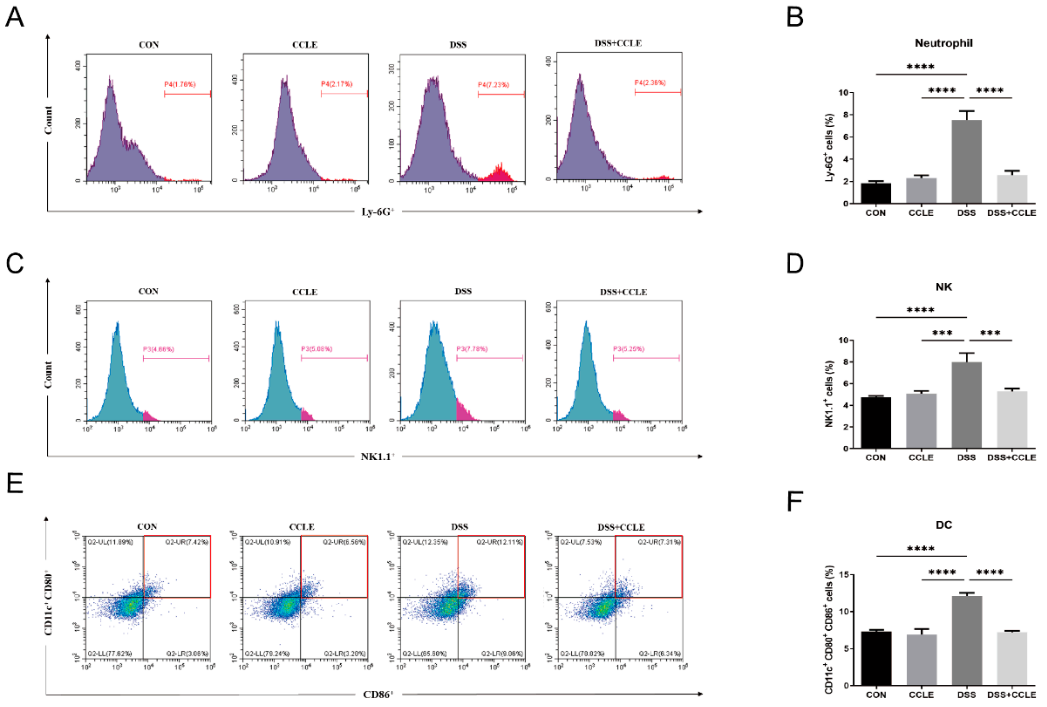

Supplemental Figure S3. Effect of CCLE on splenic immune cells of DSS-induced IBD mice. Flow cytometry analysis of (**A**) neutrophils (Ly6G+), (**C**) NK cells (NK1.1+), and (**E**) dendritic cells (CD11c+ CD80+ CD86+). Quantitative analysis of (**B**) neutrophils (Ly6G+), (**D**) NK cells (NK1.1+), and (**F**) dendritic cells (CD11c+ CD80+ CD86+) in splenic immune cells of DSS-induced IBD mice. Data are expressed as mean ± SEM (*n* = 3). ***p < 0.001, ****p < 0.0001.

II. Supplemental Tables

Supplemental Table S1. Primers for qRT-PCR.

Target Gene	Forward primer (5'→3')	Revere primer (5'→3')
IL-6	TAGTCCTTCCTACCCCAATTTCC	TTGGTCCTTAGCCACTCCTTC
TNF-α	TACTGAACTTCGGGGTGATTGGTCC	CAGCCTTGTCCCTTGAAGAGAACC
IL-1β	CCTCGTGCTGTCGGACCCATA	CAGGCTTGTGCTCTGCTTGTGA
IL-10	CTTCGAGATCTCCGAGATGCCTTC	ATTCTTCACCTGCTCCACGGC CTT
TGF-β	GAAGGCAGAGTTCAGGGTCTT	GGTTCCTGTCTTTGTGGTGAA
iNOS	GACGAGACGGATAGGCAGAG	CACATGCAAGGAAGGGAACT
ZO-1	GGGAAAACCCGAAACTGATG	GCTGTACTGTGAGGGCAACG
Occludin	ATAATGGGAGTGAACCCGACG	CGATCCATCTTTCTTCGGGTTT
SOD1	CAGGACCTCATTTTAATCCTCAC	TGCCCAGGTCTCCAACAT
CAT	GGCACACTTTGACAGAGAGCGGAT	AGTTTTTGATGCCCTGGTCGGTCT
NRF2	GCCCACATTCCCAAACAAGAT	CCAGAGAGCTATTGAGGGACTG
NQO1	TGGCCGAACACAAGAAGCTG	GCTACGAGCACTCTCTCAAACC
β-actin	AGTGTGACGTTGACATCCGTA	GCCAGAGCAGTAATCTCCTTCT
